# Atomic-Level Insights into the Adsorption of Methyl-Substituted Quinoxalinones on Fe(110): A Dispersion-Corrected DFT Analysis

**DOI:** 10.3390/molecules29215123

**Published:** 2024-10-30

**Authors:** Hassane Lgaz, Ali Aldalbahi, Han-Seung Lee

**Affiliations:** 1 Innovative Durable Building and Infrastructure Research Center, Center for Creative Convergence Education, Hanyang University ERICA, 55 Hanyangdaehak-ro, Sangrok-gu, Ansan-si 15588, Gyeonggi-do, Republic of Korea; hlgaz@hanyang.ac.kr; 2 Department of Chemistry, College of Science, King Saud University, Riyadh 11451, Saudi Arabia; aaldalbahi@ksu.edu.sa; 3 Department of Architectural Engineering, Hanyang University ERICA, 55 Hanyangdaehak-ro, Sangrok-gu, Ansan-si 15588, Gyeonggi-do, Republic of Korea

**Keywords:** corrosion inhibition, quinoxalinones, density functional theory, adsorption properties, Fe(110) surface, electron density difference

## Abstract

Corrosion of metallic equipment is a critical issue across various industries, necessitating the development of advanced protective strategies. This study utilized dispersion-corrected density functional theory (DFT) with Becke–Johnson D3(BJ) to examine the atomic-level adsorption of quinoxalinones on Fe(110) surfaces, focusing on optimizing substitution strategies to enhance corrosion inhibition. Three quinoxalinones, quinoxalin-2(1H)-one (QNO), 3-methylquinoxalin-2(1H)-one (QNOM), and 3,7-dimethylquinoxalin-2(1H)-one (QNO2M), were investigated in various configurations and protonation states. Protonated quinoxalinones demonstrated a stronger surface affinity, primarily interacting through oxygen atoms and conjugated systems, with greater energetic stability compared to neutral molecules, driven by enhanced electrostatic interactions and charge transfer mechanisms. The parallel adsorption configuration was more stable than the perpendicular mode, which in some adsorption systems did not form bonds with the iron surface. Notably, the presence of methyl substitutions did not significantly enhance adsorption strength; QNO exhibited higher energetic stability due to reduced steric interference, which maintained its planarity. Projected density of states (PDOS), electron density difference (EDD), and electron localization function (ELF) analyses confirmed the importance of charge transfer between quinoxalinone active sites and the 3d orbitals of iron in stabilizing the adsorption of molecules. These findings underscore the importance of judicious quinoxalinone functionalization to preserve their efficacy as corrosion inhibitors.

## 1. Introduction

Quinoxalinones belong to the benzodiazine heterocyclic class, characterized by a bicyclic structure comprising a benzene ring fused to a pyrazine ring [1]. The core structure features nitrogen atoms at the 1- and 4-positions, alongside a carbonyl group at the 2-position, forming the quinoxalinone framework [2]. This combination of nitrogen atoms and a carbonyl group imparts notable electron-donating and electron-withdrawing properties, which significantly influence their electronic behavior and reactivity [1,3]. These properties make quinoxalinones highly suitable for applications involving surface interactions, such as corrosion inhibition, where surface adsorption plays a crucial role [4,5]. In addition to biological and pharmacological uses, quinoxalinones’ molecular structure enables them to form stable bonds with metallic substrates, enhancing their protective capabilities in corrosion prevention.

Corrosion inhibition is one of quinoxalinones’ particular uses, where their ability to form stable complexes with metal surfaces is paramount [4]. Their electron-rich properties enable the formation of a protective layer on metal surfaces, thereby reducing electrochemical reactions that lead to corrosion [6,7]. This protective capacity is enhanced by their ability to coordinate with metals’ atoms [8,9].

Recent studies have extensively explored various quinoxaline derivatives for their corrosion inhibition properties [4]. However, despite the extensive research into their practical applications, there remains a significant gap in understanding the fundamental mechanisms of action. This gap, particularly from a theoretical perspective, offers a unique opportunity for further exploration. Investigating how the molecular structure of quinoxalinones affects surface adsorption and inhibition mechanisms at the molecular level could lead to the discovery of new compounds with improved inhibition performance.

On the other hand, reported corrosion inhibition studies rely heavily on experimental trial-and-error methodologies. Experimental techniques such as electrochemical impedance spectroscopy (EIS) and potentiodynamic polarization are routinely used to assess the performance of inhibitors by measuring their effect on metal corrosion rates in different environments [10,11]. These techniques provide valuable data on the protective capabilities of organic compounds such as quinoxalinones. However, on the opposite side, computational methods like density functional theory (DFT) offer deeper insights into the electronic properties and adsorption behavior of such inhibitors [12]. First-principles DFT calculations can effectively predict electronic characteristics and interaction energies between organic molecules and metal surfaces, enabling a better understanding of the molecular factors influencing corrosion inhibition [13,14].

A growing number of studies have successfully used computational approaches to investigate corrosion inhibition mechanisms [15]. Advanced computational tools such as ab initio DFT and DFTB (density functional tight binding) have been used to gain insights that experimental studies cannot offer [16,17]. Specifically, comprehensive analysis of projected density of states (PDOS), electron density difference (EDD) plots, and electron localization function (ELF) of adsorption systems can provide a more detailed view of molecular interactions of quinoxalinones with metal surfaces. However, a significant gap remains in this regard.

**Figure 1:**
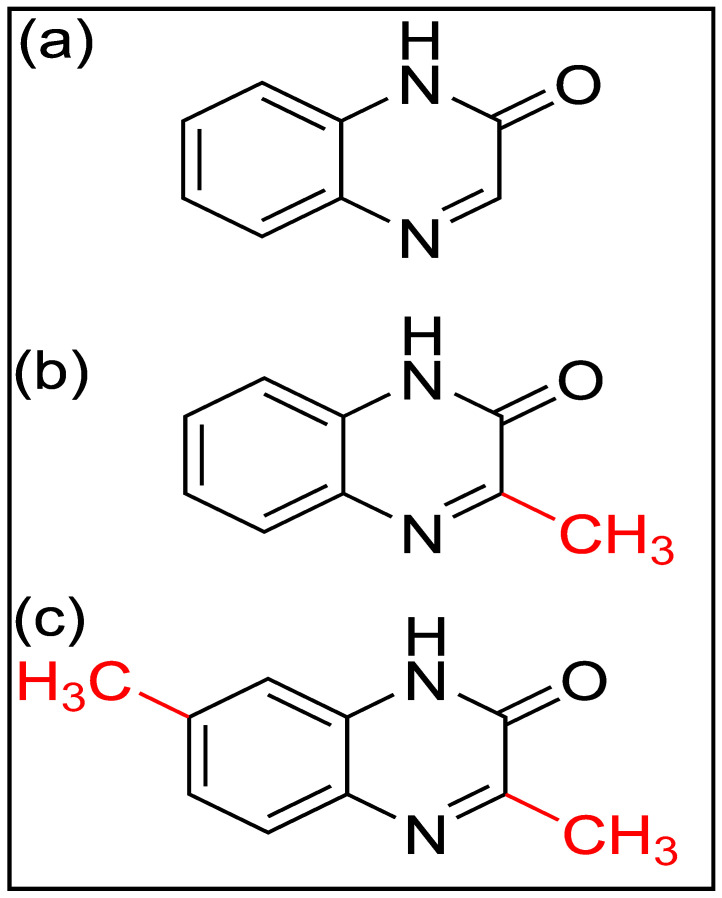
Molecular structure of investigated quinoxalinones. (**a**) QNO (quinoxalin-2(1H)-one), (**b**) QNOM (3-methylquinoxalin-2(1H)-one), and (**c**) QNO2M (3, 7-dimethylquinoxalin-2(1H)-one).

The objective of this research is to address the current gaps in understanding surface adsorption of quinoxalinones, as corrosion inhibitors for metallic equipment, at the molecular level. This study aimed to employ dispersion-corrected density functional theory (DFT) with Becke–Johnson D3(BJ) to explore the adsorption properties of three quinoxalinone derivatives, namely, quinoxalin-2(1H)-one (QNO), 3-methylquinoxalin-2(1H)-one (QNOM), and 3, 7-dimethylquinoxalin-2(1H)-one (QNO2M) (Figure 1). This research employed advanced computational modelling and analyses to uncover molecular-level mechanisms of corrosion inhibition. The ultimate aim is to aid in developing more efficient corrosion inhibitors for diverse industrial applications, ensuring that functionalization improves performance.

## 2. Results and Discussion

### 2.1. Optimized Geometries of Adsorbed Systems

The adsorption of organic molecules on metal surfaces is a key criterion for their classification as corrosion inhibitors [18,19]. Successful surface adsorption enhances the protective capacity that reduces direct metal exposure to corrosive environments, thereby slowing down the corrosion process. Quinoxalinones, with their nitrogen and oxygen heteroatoms along with the planar conjugated system, are particularly attractive as corrosion inhibitors due to their ability to form stable interactions with metal surfaces [20,21,22]. As emphasized in the literature, the effectiveness of an inhibitor is closely tied to its adsorption characteristics on the metal surface [13]. This section explores the adsorption characteristics of quinoxalin-2(1H)-one (QNO), 3-methylquinoxalin-2(1H)-one (QNOM), and 3,7-dimethylquinoxalin-2(1H)-one (QNO2M) on Fe(110), providing insights into their interaction mechanisms in neutral and protonated forms.

**Figure 2:**
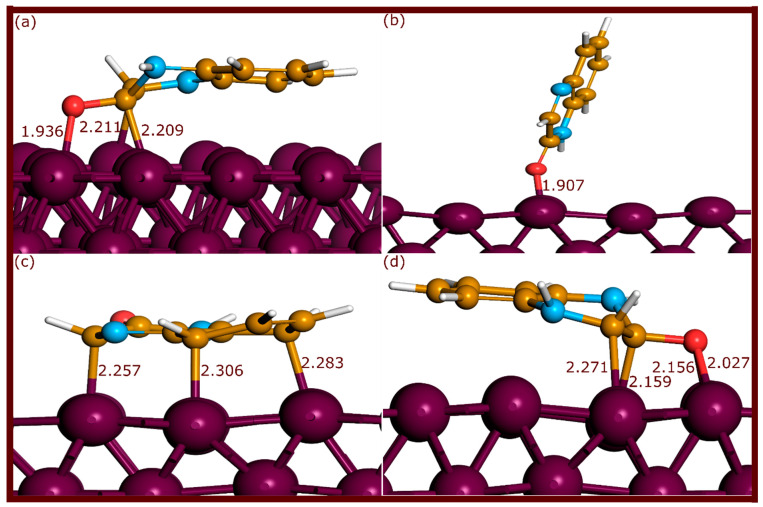
Adsorption configurations of QNO molecules on the Fe(110) surface after optimization through ab initio DFT-D3 simulations. (**a**–**d**) refer to QNO, QNO2, QNO3, and QNOH, respectively. The bond lengths are expressed in Å.

**Figure 3:**
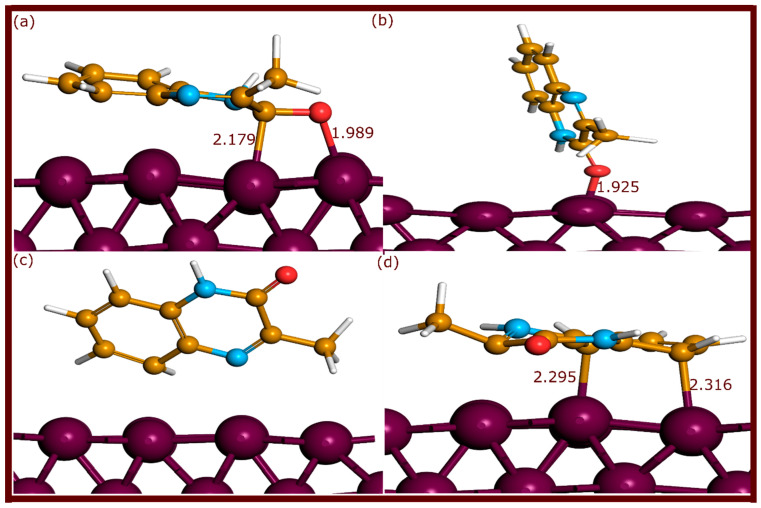
Optimized adsorption geometries of QNOM molecules on the Fe(110) surface through ab initio DFT-D3 simulations. (**a**–**d**) refer to QNOM, QNOM2, QNOM3 and QNOMH, respectively. The bond lengths are expressed in Å.

**Figure 4:**
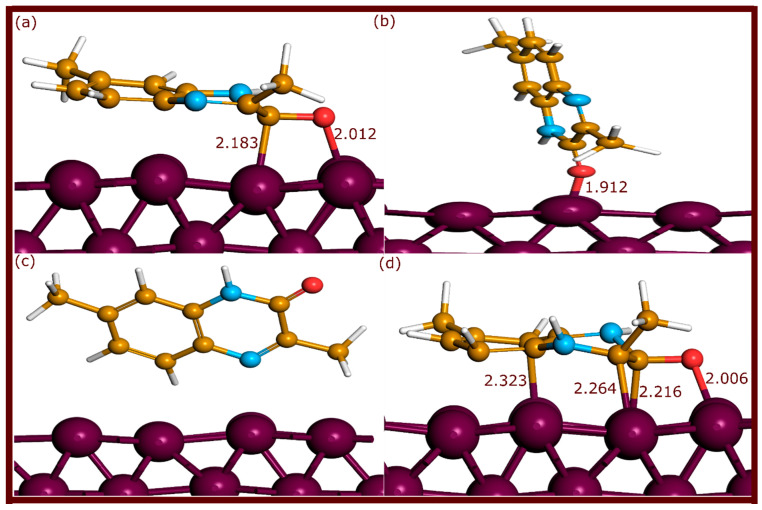
Adsorption configurations of QNO2M molecules on the Fe(110) surface after optimization through ab initio DFT-D3 simulations. (**a**–**d**) refer to QNO2M, QNO2M2, QNO2M3, QNO2MH, respectively. The bond lengths are expressed in Å.

The neutral and protonated forms of QNO, QNOM, and QNO2M exhibit different adsorption configurations on the Fe(110) surface upon optimization, with notable variations in bond lengths and coordination modes, as shown in Figure 2, Figure 3 and Figure 4.

For QNO, the neutral molecule in parallel orientation formed three strong bonds: one Fe–O bond at 1.936 Å and two Fe–C bonds at 2.209 Å and 2.211 Å, indicating strong adsorption. In the protonated state (QNOH), the molecule formed two Fe–O bonds (2.027 Å and 2.156 Å) and two Fe–C bonds (2.159 Å and 2.271 Å), slightly elongating the bond lengths. The perpendicular configuration of the quinoxalinone on the nitrogen side (QNO3) reoriented to a parallel configuration during optimization, forming three Fe–C bonds with distances of 2.283, 2.306, and 2.257 Å. This confirms that this molecule tends to be more energetically stable in its parallel form. In contrast, the perpendicular configuration on the oxygen side (QNO2) created only a single Fe–O bond at 1.907 Å, reflecting weaker adsorption.

For QNOM, the additional methyl group at position 3 caused notable changes. The neutral parallel configuration (QNOM) formed two bonds: one Fe–O at 1.989 Å and one Fe–C at 2.179 Å. The protonated form (QNOMH) formed two Fe–C bonds at 2.316 Å and 2.295 Å, indicating weaker coordination compared to QNO. The perpendicular configuration on the nitrogen side (QNOM3) did not create any bonds, while the perpendicular configuration on the oxygen side (QNOM2) formed a single Fe–O bond at 1.925 Å.

The QNO2M molecule, with two methyl groups at positions 3 and 7, showed similar trends. In the neutral parallel configuration (QNO2M), the molecule formed an Fe–O bond at 2.012 Å and an Fe–C bond at 2.183 Å. The protonated form (QNO2MH) created one Fe–O bond at 2.006 Å and three Fe–C bonds (2.323 Å, 2.264 Å, and 2.216 Å). As with QNOM3, the perpendicular configuration on the nitrogen side (QNO2M3) did not form any bonds, while the perpendicular configuration on the oxygen side (QNO2M2) formed a single Fe–O bond at 1.912 Å. In comparison with the experimental covalent radii of Fe–O (1.98 Å) and Fe–C (2.08 Å), these bond lengths suggest relatively strong metal–molecule interactions, particularly in the parallel and protonated configurations [23].

The interaction energies further corroborated the stability of the adsorption configurations. As listed in Table 1, for QNO, the neutral molecule in a parallel configuration (QNO) exhibited an interaction energy of −1.715 eV, while the protonated form (QNOH) showed a stronger interaction energy of −1.823 eV, indicating thermodynamic favorability for protonated adsorption [24,25]. Similarly, QNOM displayed stronger adsorption in the protonated state (QNOMH, −1.753 eV) compared to the neutral form (QNOM, −1.680 eV). QNO2M showed a similar pattern, with the protonated molecule (QNO2MH) having an interaction energy of −1.743 eV compared to the neutral parallel configuration (QNO2M, −1.669 eV).

The comparison across the three molecules suggests that protonation generally enhances adsorption strength, as seen in the more negative interaction energies for the protonated forms. This can be attributed to the strong contribution of long-range van der Waals interactions to the stability of molecules under study. However, the effect of additional methyl groups on QNOM and QNO2M did not significantly increase interaction energy compared to QNO. This observation highlights a potential limitation in methylation’s influence on adsorption energy, suggesting that while methyl groups may impact steric factors, they do not substantially improve bonding. Interestingly, it can be stated that methylation alters the adsorption strength of the parent molecule, which is well reflected in the adsorption configurations of QNOM3 and QNO2M3, where no bonding is observed compared to strongly adsorbed QNO3. The core quinoxalinone is the main factor responsible for coordination with the Fe surface, and therefore each molecule’s bonding characteristics. Notably, the perpendicular configurations showed weaker adsorption, attributed to the preferred parallel adsorption mode of this kind of molecules [26]. The QNO molecule seemed to be in an optimal adsorption mode without additional functionalization.

The results from this section provide crucial insights for the further design of quinoxalinones. The findings emphasize the critical effect that a small functional group may have on the adsorption characteristics. Given the present investigation and its conditions, it can be concluded that the overall impact of the present functionalization strategy on adsorption energy is limited. In contrast, the protonation state significantly improved adsorption stability. It is well-reported that protonated molecules play an important role in the initial adsorption process of inhibitors, specifically in acidic media [27,28].

### 2.2. Projected Density of States

Projected density of states (PDOS) analysis is a vital tool for examining metal–molecule interactions, particularly in the context of corrosion inhibition [17,29]. By decomposing the total electronic structure into contributions from individual atomic orbitals, PDOS allows us to identify orbital interactions that are responsible for stabilizing adsorption complexes. This approach provides detailed insight into how specific orbitals of the organic molecules hybridize with the Fe surface, leading to charge transfer and bonding [17].

**Figure 5:**
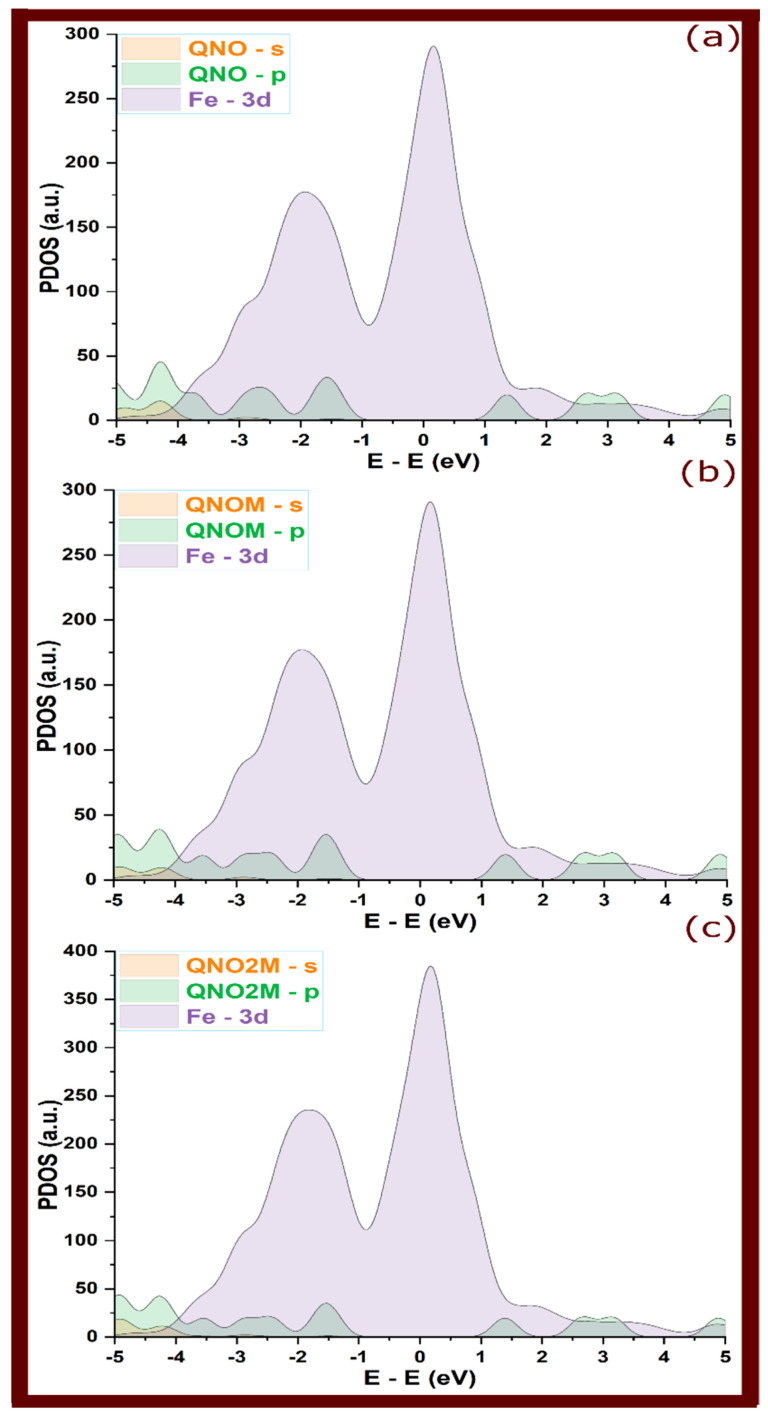
Projected density of states for quinoxalinone derivatives at their isolated state, situated 6 Å above the top layer of the iron surface; (**a**–**c**) refer to QNO, QNOM, and QNO2M, respectively.

As shown in Figure 5, in their isolated states, placed 6 Å above the Fe(110) surface to minimize interaction, the PDOS for the quinoxalin-2(1H)-one (QNO), 3-methylquinoxalin-2(1H)-one (QNOM), and 3,7-dimethylquinoxalin-2(1H)-one (QNO2M) molecules exhibited distinct peaks within the range of −5 eV to 5 eV [30]. These states align closely with the unoccupied Fe 3d states. This suggests that when the molecules adsorb onto the surface, significant orbital hybridization might occur. This alignment between the molecular orbitals and the metal’s 3d orbitals indicates that these states are likely to participate in bonding, enhancing the interaction strength upon adsorption.

**Figure 6:**
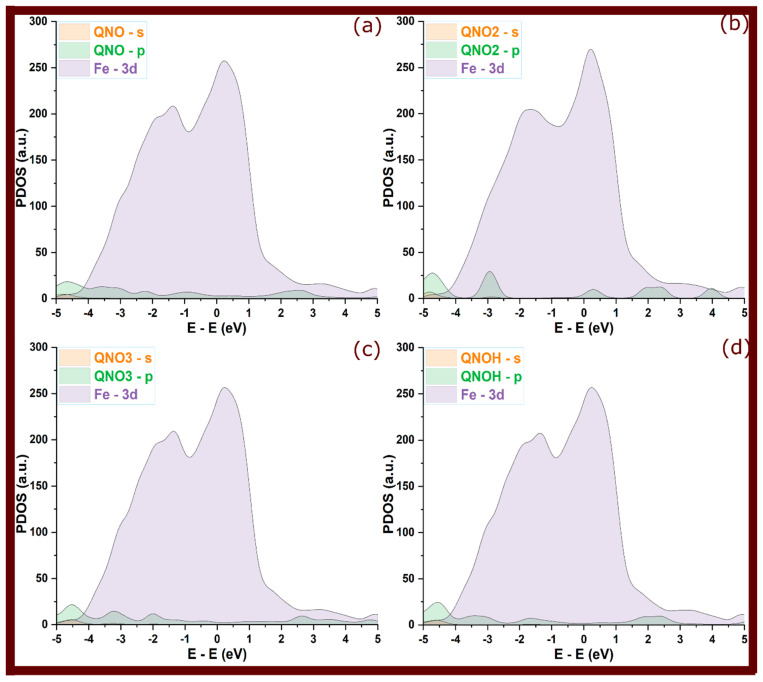
Projected density of states for QNO quinoxalinone derivative at its adsorbed state; (**a**–**d**) refer to QNO, QNO2, QNO3, and QNOH, respectively.

**Figure 7:**
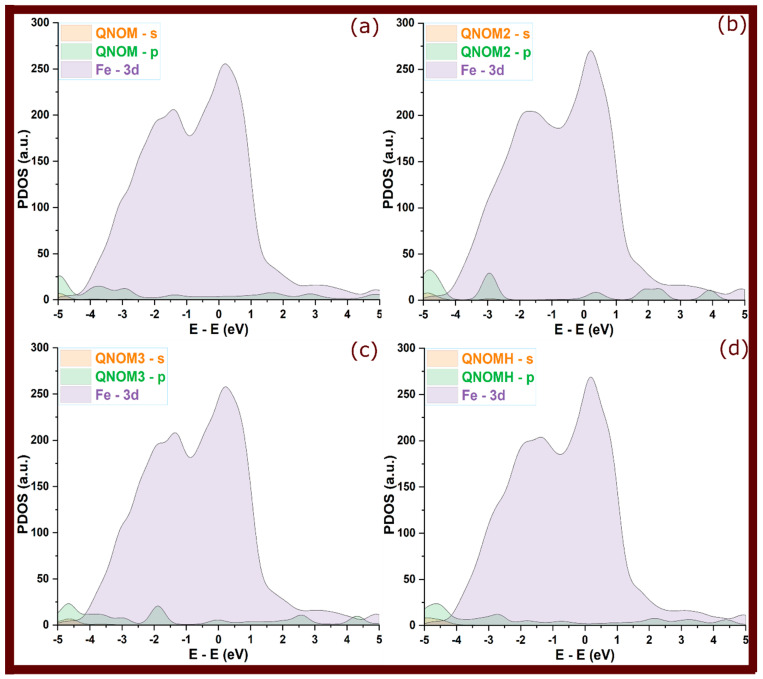
Projected density of states for QNOM quinoxalinone derivative at its adsorbed state; (**a**–**d**) refer to QNOM, QNOM2, QNOM3, and QNOMH, respectively.

**Figure 8:**
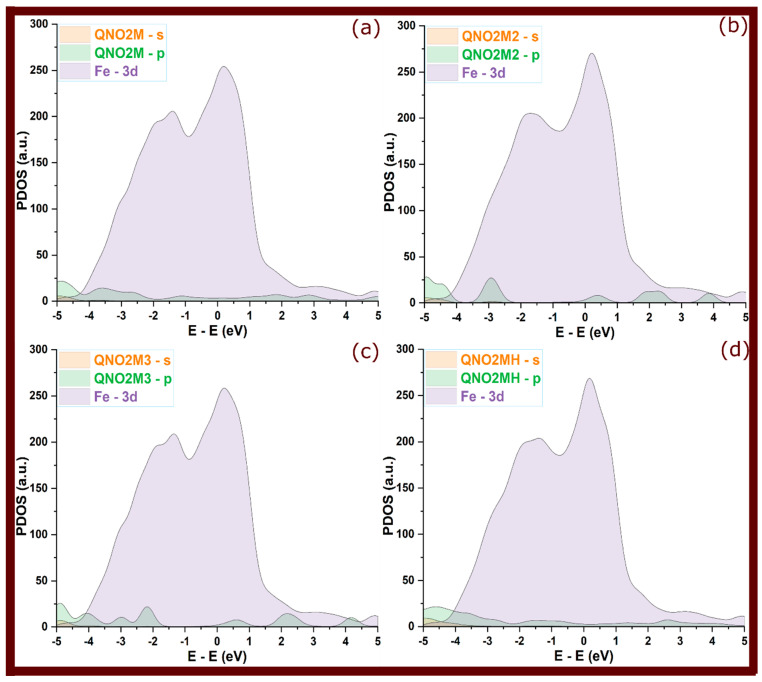
Projected density of states for QNO2M quinoxalinone derivative at its adsorbed state; (**a**–**d**) refer to QNO2M, QNO2M2, QNO2M3, and QNO2MH, respectively.

So, in Figure 6, Figure 7 and Figure 8 of adsorbed molecules, upon adsorption of quinoxalinone molecules onto the Fe(110) surface, the PDOS showed clear signs of electronic interaction. The sharp peaks observed in the isolated state were significantly broadened and attenuated. This broadening reflects an increased electronic disorder due to interaction with the metal surface, signaling orbital overlap between the molecules and the metal [31,32]. Specifically, the s and p orbitals of the quinoxalinones hybridized with the Fe 3d orbitals, reinforcing the covalent bonding character. The broadening of molecular states, particularly those around the Fermi level (0 eV), indicates that electrons from the molecule’s occupied states were delocalized over the metal, suggesting strong adsorption [17]. However, perpendicular configurations that exhibited weaker interactions (as seen in some QNOM and QNO2M cases) showed less pronounced broadening, indicating weaker bonding and minimal orbital overlap in these orientations.

The hybridization of the molecules’ s and p orbitals with the metal’s 3d states is a key mechanism that stabilizes the adsorbed complexes. PDOS analysis suggests that charge transfer occurs from the molecule to the metal, particularly in the case of protonated molecules, where greater orbital overlap is observed. This transfer is evident in the redistribution of electronic states near the Fermi level, indicating covalent bonding and some degree of electron sharing. The presence of such covalent interactions enhances the inhibitor’s performance by effectively minimizing the electrochemical reactions responsible for corrosion.

While PDOS provides crucial insights into orbital interactions and bonding mechanisms, a more comprehensive understanding of the adsorption process requires additional electronic structure analyses. EDD and ELF analyses are also reported to complement the PDOS findings by visualizing charge redistribution and electron localization.

### 2.3. Electron Density Difference Analysis

EDD analysis serves as a critical complement to PDOS when studying the electronic interactions between organic molecules and metal surfaces. While PDOS provides insights into orbital interactions, EDD plots visualize charge redistribution between the adsorbed molecules and the metal surface. These maps display regions of electron accumulation (colored red) and electron depletion (colored yellow), highlighting areas where bonding occurs and charge transfer takes place.

**Figure 9:**
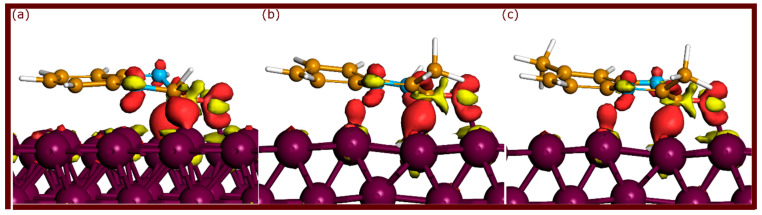
EDD plots for quinoxalinones adsorbed on the Fe(110) surface, with an isosurface value of 0.05 e/Å^3^. (**a**) QNO, (**b**) QNOM, and (**c**) QNO2M.

In this analysis, the three parallel adsorption configurations of neutral quinoxalinones were considered for comparison. The EDD plots are shown in Figure 9. This figure reveals distinct patterns of charge redistribution. Significant electron accumulation (red) was observed around the Fe-C bonds, indicating strong interaction between the π-conjugated systems of the molecule and the metal surface. In particular, the Fe-O bond also showed considerable electron accumulation, emphasizing the role of oxygen as a key site for interaction with iron. The electron depletion regions (yellow) were primarily located around the areas between the molecular core and the metal surface, suggesting electron flow from the molecules to the metal. This redistribution indicates a strong quinoxalinone–Fe interaction, further solidifying the role of these bonds in stabilizing the adsorbed state [33,34].

The EDD plots show evidence of a dual charge transfer mechanism at the molecule–metal interface. This is attributed to electron donation from the active sites of the organic molecules, particularly π-systems and oxygen atoms, into the vacant d-orbitals of the Fe surface. Simultaneously, back-donation occurs from the filled d-orbitals of the metal to the π* orbitals of the molecules, particularly in the conjugated ring systems [12,15]. This bidirectional charge transfer strengthens the covalent bonding between the molecules and the metal. The extent of charge accumulation around the Fe-C and Fe-O bonds is indicative of the strength of these covalent interactions, with larger red regions corresponding to stronger electron sharing between the molecules and metal.

The findings from the EDD analysis confirm that strong covalent interactions and charge transfer mechanisms stabilize the adsorption of quinoxalinones on the Fe(110) surface. These results are consistent with PDOS findings, where hybridization of molecular orbitals with metal d-states reinforces the strength of interactions.

### 2.4. Electron Localization Function Maps

The ELF is a powerful tool for analyzing the electronic configurations and bonding characteristics of molecules adsorbed on metal surfaces [35,36]. ELF provides a visual representation of electron localization, allowing for the differentiation between different types of chemical bonding. High ELF values, approaching 1, indicate strong covalent bonds where electrons are highly localized, typically in areas with shared electron pairs. Values around 0.5 represent electron delocalization, suggesting weaker, metallic, or physisorptive interactions. Lower ELF values below 0.5 are indicative of metallic bonding or weaker van der Waals forces, typical in physisorption. By mapping these ELF values, one can visually distinguish between strong chemisorption and weaker physisorption [35].

**Figure 10:**
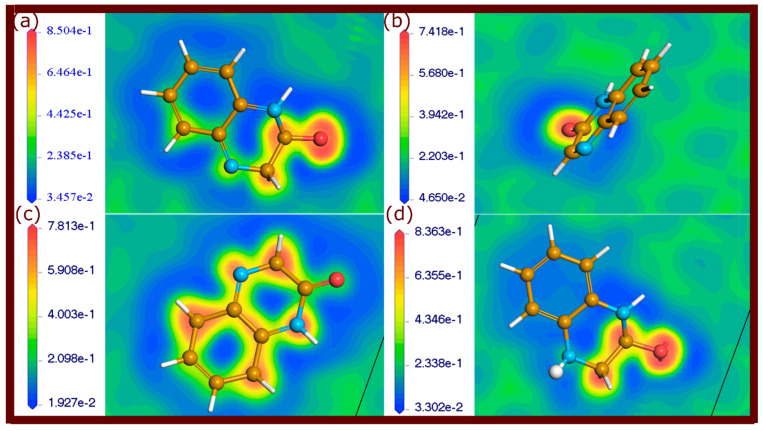
ELF plots of QNO molecules adsorbed on the Fe(110) surface, illustrating electron localization. (**a**) QNO, (**b**) QNO2, (**c**) QNO3, and (**d**) QNOH.

**Figure 11:**
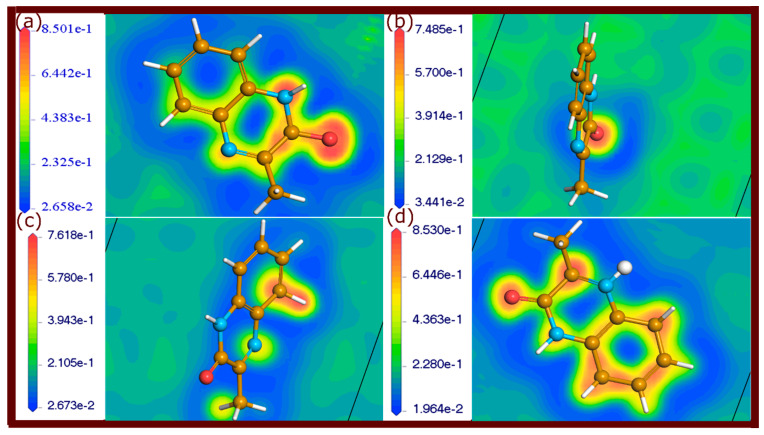
ELF plots of QNOM molecules adsorbed on the Fe(110) surface, illustrating electron localization. (**a**–**d**) represents QNOM, QNOM2, QNOM3, and QNOMH, respectively.

**Figure 12:**
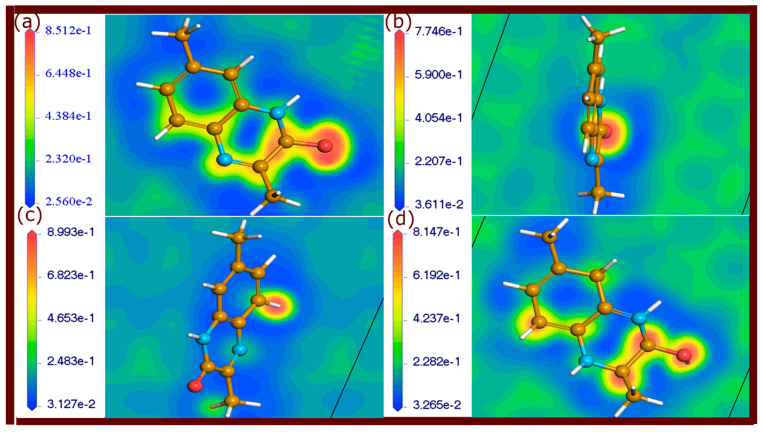
ELF plots of QNO molecules adsorbed on the Fe(110) surface, illustrating electron localization. (**a**–**d**) represents QNO2M, QNO2M2, QNO2M3, and QNO2MH, respectively.

ELF maps of the neutral and protonated forms of investigated quinoxalinones adsorbed on the metal surface are shown in Figure 10, Figure 11 and Figure 12. Different regions of electron localization are color-coded. In these figures, red areas indicate regions of high electron localization, suggesting strong covalent bonding, particularly near the oxygen atoms of carbonyl groups where Fe-O bonds are present. The red-yellow regions represent partial electron localization, indicative of weaker physisorption interactions, which are located around the aromatic rings and nitrogen atoms. Yellow-green regions are observed where weaker interactions or metallic bonding occurs, signifying less localized electron density. These maps reveal that electron-rich areas around oxygen atoms and some carbon atoms contribute to strong covalent bonds with the metal. It should be noted that some π-electrons of aromatic rings are involved in covalent and non-covalent interactions.

The ELF plots provide clear insights into the chemical bonding characteristics at the molecule–metal interface. The physisorptive interactions, although weaker, complement the stronger covalent bonds, providing additional stability.

The findings from the ELF analysis demonstrate that a combination of strong covalent bonds and physisorption bonding enhances the stability of the adsorbed molecules on the metal surface. The presence of highly localized electron density in certain regions ensures strong chemisorptive interactions, while the weaker physisorptive bonds provide flexibility and adaptability at the molecule–metal interface. The ELF analysis thus confirms the robust interaction between the quinoxalinone molecules and the metal surface.

## 3. Computational Details

### 3.1. DFT Computation Details

**Figure 13:**
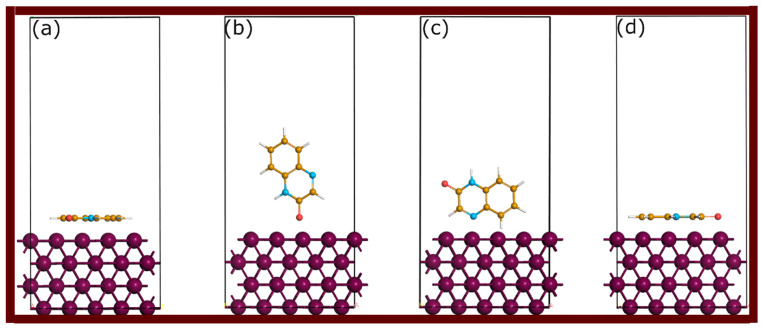
Initial adsorption configurations for the adsorption of QNO on the Fe(110) surface. (**a**) QNO, (**b**) QNO2, (**c**) QNO3, and (**d**) QNOH. The same abbreviations were used for QNOM (QNOM, QNOM2, QNOM3, and QNOMH) and QNO2M (QNO2M, QNO2M2, QNO2M3, and QNO2MH).

The first-principles simulations were performed using the CASTEP software package implemented in Materials Studio 7.0, which is specifically designed for material simulations based on density functional theory (DFT) [37]. Spin-polarized DFT was employed to account for magnetic effects in the system. The DFT method, with the Becke–Johnson D3(BJ) dispersion-corrected technique, was applied to include van der Waals interactions [38,39]. The generalized gradient approximation (GGA) was used to treat exchange-correlation, specifically the Perdew–Wang functional [40,41]. A plane-wave basis set was used with a kinetic energy cut-off of 30 Ry. To ensure convergence of the calculations, a self-consistent field (SCF) tolerance of 1 × 10⁻⁶ eV per atom was set. The geometric structures were optimized using the Broyden–Fletcher–Goldfarb–Shanno (BFGS) algorithm, which is widely known for its reliability in molecular geometry optimization. Convergence criteria for structural optimization were set to “fine” quality within the CASTEP settings, which included stringent thresholds for force, stress, and displacement to guarantee accurate geometries. For the bulk Fe metal structure, the body-centered cubic (BCC) lattice was modeled with a Monkhorst–Pack k-point grid of 8 × 8 × 8 to sample the Brillouin zone. The Fe(110) surface was chosen to represent the iron surface because Fe is the primary component of steel responsible for interactions with inhibitor molecules in corrosion studies [33,42]. When simulating adsorption systems, the k-point grid was reduced to 2 × 2 × 1 for efficiency, as a large supercell was employed. The calculated lattice parameter for the bulk metal was 2.854 Å, closely matching the experimental value of 2.866 Å, ensuring the accuracy of the used settings prior to modelling surface interactions with quinoxalinones. Adsorption studies were conducted on the (110) surface of the iron, modelled using a slab consisting of four atomic layers in a 4 × 4 supercell. To reduce computational complexity, the optimization process kept the two bottom layers of the slab fixed, while the remaining layers were free to relax. A vacuum gap of 20 Å was introduced to eliminate interactions between periodic images. Quinoxalinone molecules were placed in various adsorption configurations, including one parallel orientation, two perpendicular orientations, and one protonated parallel mode, to reflect common adsorption behavior. Initial configurations and their abbreviations are represented in Figure 13. To simulate isolated molecules, the “*molecule in a box*” approach was applied. The simulation box was set to 40 Å in each dimension to prevent artificial interactions between the molecule and its periodic images.

### 3.2. Interaction Energies and Electronic Characteristics

The interaction energy ($E_{inter}$) quantifies the strength of the interaction between the quinoxalinone molecule and the Fe(110) metal surface. It is calculated by subtracting the energies of the isolated molecule and metal surface from the total energy of the adsorbed system. The formula is given as

(1) $$E_{inter}=E_{mol/surf}\;-\;\left(E_{mol}\;+\;E_{surf}\right)\;\;$$

where $E_{mol/surf}$ is the total energy of the molecule–metal system, $E_{mol}$ is the energy of the standalone molecule, and $E_{surf}$ is the energy of the isolated metal surface. A negative value for $E_{inter}$ indicates a stable adsorption configuration.

To investigate charge transfer between the molecule and the metal surface, charge density difference ($\Delta \rho \left(r\right)$) calculations were performed. This provides a spatial representation of electron redistribution upon adsorption. The charge density difference is calculated as

(2) $$\Delta \rho \left(r\right)=\rho_{\frac{mol}{Fe\left(110\right)}}\left(r\right)-\rho_{Fe\left(110\right)}\left(r\right)-\rho_{mol}\left(r\right)$$

where $\rho_{\frac{mol}{Fe\left(110\right)}}\left(r\right)$ is the charge density of the combined molecule–metal system, $\rho_{Fe\left(110\right)}\left(r\right)$ is the charge density of the isolated metal surface, and $\rho_{mol}\left(r\right)$ is the charge density of the isolated molecule. This calculation reveals areas of electron accumulation and depletion, highlighting charge transfer and interaction strength between the molecule and the metal.

The electron localization function (ELF) is used to analyze the bonding characteristics within the adsorption system by evaluating the likelihood of electron pair localization. The ELF is a dimensionless quantity that ranges from 0 to 1, where values close to 1 indicate high electron localization, and values near 0 suggest delocalized electrons. The ELF is computed using the following formula:

(3) $$ELF=\frac{1}{1+{\left(D\left(r\right)/Dh\left(r\right)\right)}^{2}}$$

where $D\left(r\right)$ represents the electron localization at position r, and $Dh\left(r\right)$ is the localization function for a homogeneous electron gas. ELF helps visualize regions of strong bonding and local electron concentration in the molecule–metal system, offering insight into the nature of chemical bonds formed during adsorption.

The PDOS was computed to investigate the interaction of molecular orbitals with the metal surface. PDOS was calculated for the isolated molecule positioned 6 Å above the top layer of the metal surface, as well as for the adsorbed molecule. For iron, the 3d orbitals were considered, while for the organic molecule, the s and p orbitals were analyzed. This comparison allowed us to understand how molecular states hybridize with metal states during adsorption, identifying specific orbital contributions to the bonding.

Together, these analyses elucidate the electronic structure, bonding mechanisms, and charge redistribution during quinoxalinone adsorption on the Fe(110) surface.

## 4. Conclusions

This work reported the ab initio DFT computation of the adsorption characteristics of quinoxalin-2(1H)-one and two methyl substituted quinoxalin-2(1H)-ones on the Fe(110) surface. A DFT with the D3 dispersion correction methodology was employed to assess the bonding behavior, interaction energies, and electronic properties of quinoxalinones under different adsorption configurations and the protonation state. The following conclusions can be drawn from this study:

- Adsorption of quinoxalinones on Fe(110) showed stable interaction energies, with protonated forms exhibiting stronger adsorption than neutral ones.
- Charge transfer between the molecule and metal surface played a key role in adsorption stability, particularly through electron donation from the molecule’s active sites to the metal’s d-orbitals and back-donation to the π*-orbitals.
- EDD analysis highlighted significant electron accumulation (red) around Fe-C and Fe-O bonds, showing strong covalent interactions.
- ELF analysis showed strong covalent bonding (high electron localization) near oxygen atoms and regions of Fe-C bonding, confirming robust chemisorption.
- Physisorption was also found to contribute significantly through nitrogen atoms and aromatic rings, enhancing adsorption stability via weaker interactions.
- PDOS analysis revealed molecular orbital hybridization with metal d-orbitals, with the broadening of molecular peaks upon adsorption, indicating stronger molecule–metal interaction.

The stable adsorption of quinoxalinones on Fe(110) is reinforced by a combination of strong covalent bonds and physisorption, enhancing their effectiveness as corrosion inhibitors. The interplay between chemisorption and physisorption can form a protective layer, which is essential for preventing corrosion.

## Figures and Tables

**Table 1:** Interaction energies for the adsorption of quinoxalinone derivatives on the Fe(110) surface.

Molecule	Interaction Energy (in eV)	Molecule	Interaction Energy (in eV)	Molecule	Interaction Energy (in eV)
QNO	−1.715	QNOM	−1.680	QNO2M	−1.669
QNO2	−1.514	QNOM2	−1.508	QNO2M2	−1.491
QNO3	−1.683	QNOM3	−1.541	QNO2M3	−1.530
QNOH	−1.823	QNOMH	−1.753	QNO2MH	−1.743

## Data Availability

The data presented in this study are part of an ongoing study and cannot be shared at this time.
